# LC3A Silencing Hinders Aggresome Vimentin Cage Clearance in Primary Choroid Plexus Carcinoma

**DOI:** 10.1038/s41598-017-07403-5

**Published:** 2017-08-14

**Authors:** Marwa Nassar, Heba Samaha, Myret Ghabriel, Maha Yehia, Hala Taha, Sherin Salem, Khaled Shaaban, Mariam Omar, Nabil Ahmed, Shahenda El-Naggar

**Affiliations:** 1 Tumor Biology Research Program, Basic Research Unit, Department of Research, Children’s Cancer Hospital Egypt 57357, P.O Box 11441 1 Seket Al-Emam Street, Cairo, Egypt; 2 Department of Pathology, Children’s Cancer Hospital Egypt 57357, P.O Box 11441 1 Seket Al-Emam Street, Cairo, Egypt; 3 Department of Clinical Pathology, Children’s Cancer Hospital Egypt 57357, P.O Box 11441 1 Seket Al-Emam Street, Cairo, Egypt; 4 Biotechnology Graduate Program, American University in Cairo. New Cairo Campus, AUC Avenue, P.O Box 74, New Cairo, 11835 Egypt; 5 Center for Cell and Gene Therapy, Texas Children’s Hospital, Baylor College of Medicine, 1102 Bates St. Suite 1700, Houston, Texas 77030 USA; 6 National Cancer Institute (NCI), Cairo, Egypt

## Abstract

Aggresomes are transient microtubule-dependent inclusion bodies that sequester misfolded proteins and are ultimately removed by autophagy. Here we report the generation of a choroid plexus carcinoma cell line; Children’s Cancer Hospital Egypt (CCHE)-45, which is characterized by the constitutive formation of aggresomes. When examining the autophagy pathway as the main route for aggresomes clearance, CCHE-45 cells displayed increased autophagy flux mediated by MAP1LC3B. *MAP1LC3A*-Variant1 gene expression was silenced by promoter methylation. Restoring *MAP1LC3A*-Variant1 expression resulted in the formation of MAP1LC3A positive autophagosmes and the disruption of the aggresomes' vimentin cage independent of MAP1LC3B positive autophagosomes. Our data supports the notion that basal quality control autophagy and vimentin cage clearance in CCHE-45 are mediated by MAP1LC3A. Hence we propose that absence of MAP1LC3A disrupts the autophagic pathway and leads to the failure of aggresome vimentin cage degradation. Consequently, this could represent a targetable pathway in autophagy-dependent cancers.

## Introduction

Mutations, metabolic challenges, and cellular stress conditions are common reasons for the production of aberrant proteins^1^. Consequently, cells employ several quality control strategies aimed at refolding^2^, degrading or sequestering aberrant protein species^3, 4^. Insoluble protein deposit (IPOD) and intranuclear quality control (INQ) are two compartments for sorting and sequestration for cytoplasmic and nuclear proteins, respectively^5^. Aggresomes are specialized, cytoplasmic cage-like structures formed by the collapse of the intermediate filament vimentin at the microtubule organizing centers (MTOC). They sequester misfolded proteins, to be ultimately removed by autophagy^6^. Hence, aggresomes play a cytoprotective role that helps cells handle proteotoxic stress.

Autophagy was initially characterized as a non-selective cellular degradation mechanism that is initiated by nutrient deprivation^7^. Autophagy is mainly concerned with recycling long lived cellular proteins, macromolecules and damaged organelles^7^. Genetic screens in *Saccharomyces cerevisiae* have identified 31 autophagy related genes (*Atg*) that regulate the sequential steps required for the formation of autophagsomal structure to the final fusion with the lysosome. One of the main components of the autophagosome membrane is the Atg 8 protein which resides on both the inner and outer sides of the autophagosome membrane^8, 9^. In humans four Atg 8 orthologs have been identified; microtubule associated protein 1 light chain 3 (*MAP1LC3*) genes; hereafter referred to as *LC3A, LC3B, LC3Ba* and *LC3C* ^8^. In recent years LC3A was speculated to play a role in cancer^10, 11^. Here we investigate the role of autophagy in aggresome clearance in choroid plexus carcinoma tumors (CPCTs). CPCTs are rare neoplasms of the central nervous system, with 20% of tumors occurring during the first year of life^12, 13^. These patients generally have poor outcomes due to limited therapeutic options^5, 14^.

In the current study, we established a primary choroid plexus carcinoma (CPC) tumor line, CCHE-45 which constitutively formed aggresomes. CCHE-45 cells displayed disrupted autophagy flux mediated by LC3B. The lysosome inhibitor chloroquine was unable to block this flux, thus supporting altered autophagy. On the other hand, *LC3A* variant 1(*LC3A-V1*) was silenced by promoter methylation. Re-expression of *LC3A-V1* resulted in the disruption of aggresome vimentin cage, independent of the formation of LC3B autophagosomes. Taken together, the data supports a role for LC3A in quality control autophagy. Moreover, results suggest that *LC3A* gene silencing in CPC primes cells for aggresome formation to achieve cellular homeostasis, hence highlighting the role of aggresomes as a survival mechanism for cancer cells.

## Results

### HDAC6 inhibitor represses constitutive formation of aggresomes in choroid plexus carcinoma line CCHE-45

**Figure 1:**
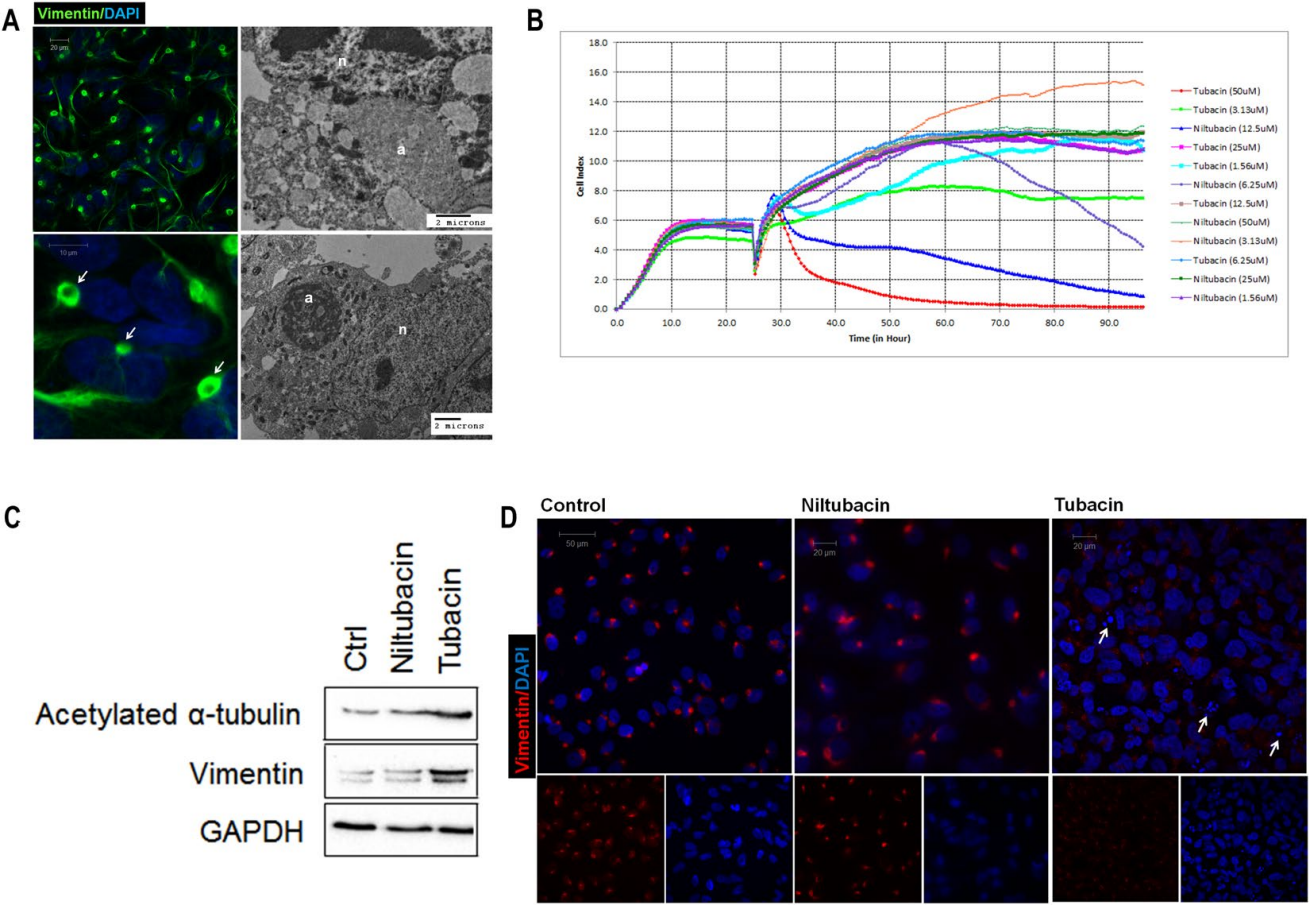
Constitutive formation of aggresomes in choroid plexus carcinoma tumor cell line CCHE-45. (**A**) Aggresomes subcellular localization was identified by the formation of vimentin cage (white arrows). CCHE-45 cells were fixed and immunostained with rabbit anti-vimentin and visualized using Alexa Flour 488 anti-rabbit IgG antibody. Cells were counterstained with DAPI to visualize the nucleus. TEM examination of CCHE-45 cell line showing aggresomes ultra structures. (**B**) The effect of tubacin and niltubacin on CCHE-45 cell line was evaluated using xCELLigence system. Cells were treated with different concentration of tubacin or niltubacin and dynamically monitored for 72 hours. Cell index was used to assess changes in cell growth under different concentrations of tubacin or niltubacin. The e xperiment was repeated three times. (**C**) Western blot analysis of CCHE-45 cells treated with tubacin or niltubacin for 24 hours or left untreated (Ctrl). GAPDH was used as a loading control. (**D**) Immunofluorescence analysis of CCHE-45 cells. Cells were treated with niltubacin, tubacin or left untreated (control) for 24 hours. Cells were immunostained with mouse anti-vimentin and counterstained using DAPI. White arrows in CCHE-45 tubacin treated cells indicate fragmented nuclei. a = aggresomes, n = nucleus, Ctrl = control﻿.

We propagated a primary cell line CCHE-45, from CPC surgical excision sample. CCHE-45 cells presented with two clones, one clone was triploid (62~75 chromosomes) and the second clone was hexaploid (137 chromosomes). Structural abnormalities in both clones included translocations (2;18)(q32;q23), (1;3)(?;q27) and (20;22)(p11;q11), and del(17) (p11) (Figure S1A). Only the hexaploid clone had two copies of each translocation. When immunostained for vimentin, a marker for CPT, CCHE-45 cells displayed a single perinuclear vimentin positive inclusion in all cells, which varied in intensity and size (Fig. 1A). The presence of vimentin cage-like structures is characteristic of aggresomes^15^. Examination of CCHE-45 cells by transmission electron microscopy (TEM) confirmed the presence of dense to light aggresomes, 2–3 µm in diameter (Fig. 1A). Juxta Nuclear Quality control compartment (JUNQ) describes vimentin-positive structures that share similar cellular positions as aggresomes^16^, and it was proposed that aggresomes may represent a mature state of JUNQ^3^. In the case of CCHE-45 cells, their constitutive presence in all cells and lack of mobility supports aggresome description rather than JUNQ. Furthermore, both CCHE-45 cells and the parent tumor displayed similar structures (Figure S1B).

In contrast to previous reports^15, 17^, cytokeratin also contributed to the structure of aggresomes (Figure S1B). Examination of cytokeratin and vimentin pattern in choroid plexus papilloma (CPP) and atypical choroid plexus papilloma (ACPP) confirmed the absence of aggresomes in these two tumor subtypes (Figure S1C). Misfolded or aggregated proteins that cannot be eliminated by the proteasome are concentrated by HDAC6 and transported by the action of the dynein motor protein to the aggresomes^6, 18^. In this context, we evaluated the effect of different concentrations of the HDAC6 inhibitor tubacin and its inactive analog niltubacin on CCHE-45 cells for 72 hours. Significant reduction in CCHE-45 cell index, which reflects changes in cell adherence, was reported in tubacin treated cells with no change in niltubacin treated cells (Fig. 1B). Due to observed effect of tubacin on CCHE-45 cell proliferation, we hypothesized that it could prevent the accumulation of aggresomes. Accordingly, CCHE-45 cells were treated with either tubacin or niltubacin for 24 hours. An increase in the levels of acetylated α-tubluin was observed following tubacin treatment, hence confirming the inhibition of HDAC6 (Fig. 1C). However, no change in vimentin was detected (Fig. 1C)^6^. Therefore, change in aggresomes’ vimentin cage was examined by immunofluorescence. CCHE-45 cells treated with tubacin presented with dissociated vimentin cage compared to niltubacin treated or control non-treated cells. Nevertheless, intact aggresomes could be detected and fragmented nuclei were observed in tubacin treated cells (Fig. 1D).

### Autophagy flux mediated by LC3B is not blocked by the lysosomal inhibitor chloroquine in CCHE-45 cells

**Figure 2:**
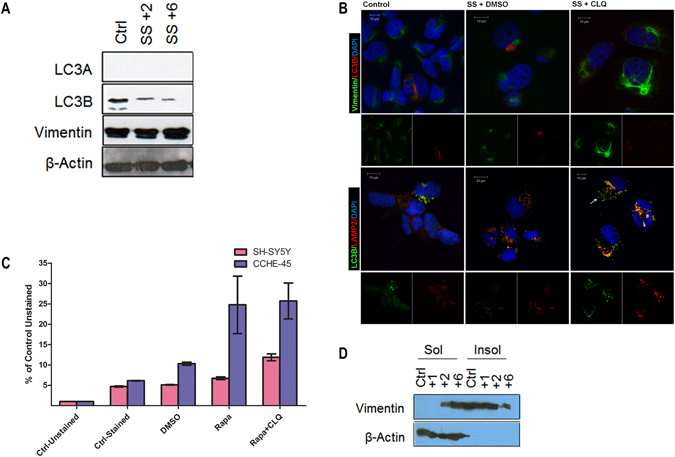
Increased autophagy flux in CCHE-45 cells is not blocked by lysosome inhibitor chloroquine. (**A**) Western blot analysis of CCHE-45 cells cultured under normal condition or serum starved for 2 or 6 hours in HBSS. β-actin was used as a loading control. (**B**) Immunofluorescence staining of CCHE45 cells. Cells were cultured under normal condition or serum starved in HBSS for 5 hours or serum starved and treated with 50 µM chloroquine. CCHE-45 cells were immunostained with rabbit anti-vimentin (green) and mouse anti-LC3B (red), or rabbit anti-LC3B (green) and mouse anti-LAMP2 (red) and visualized using Alexa Fluor 488 goat anti-rabbit antibody or Alexa Fluor 555 goat anti-mouse. White arrow heads show LC3B positive autophagosomes. (**C**) Flow cytometry-based profiling of CYTO-ID Autophagy detection for CCHE-45 and SH-SY5Y cells. Mean fluorescent intensity comparison between CCHE-45 and SH-SY5Y is representative of three independent experiments. Statistical analysis was performed using paired student’s t test. The level of significance was set at p-value of 0.05. Error bars represent average ± SEM. (**D**) Western blot analysis of soluble and insoluble protein fractions collected from CCHE-45 cells cultured under normal conditions or treated with HBSS for 1, 2 or 6 hours. Ctrl = control, SS = serum starved, Rapa = rapamycin, CLQ = chloroquine, Sol = soluble protein fraction, Insol = insoluble protein fraction.

While aggresomes formation is considered a cytoprotective mechanism, they are ultimately eliminated by autophagy^5^. LC3B is commonly used as a marker for induction of autophagy^15^; however MAP1LC3/LC3 family members include LC3A, LC3B and LC3C, where the former two were reported to participate in autophagosome formation^16, 17^. To assess the role of autophagy in aggresome clearance, CCHE-45 and SH-SY5Y cells were serum-starved in HBSS for 2 and 6 hours. After 2 hours of serum starvation, autophagic vacuoles were detected in both lines (Figure S2A) hence supporting the induction of autophagy. Furthermore, LC3B and LC3A levels were reduced in SH-SY5Y cells (Figure S2B). Similarly, CCHE-45 showed reduction in LC3B levels and no detectable LC3A in control with no change in vimentin (Fig. 2A). Autophagy flux was then examined in both lines using chloroquine to block the fusion between autophagosomes and lysosomes. LC3B puncta were detected under normal growth conditions (Fig. 2B). Following serum starvation, LC3B positive autophagsomes were found in close proximity to aggresomes, co-localizing with LAMP2 in CCHE-45 cells (Fig. 2B). Chloroquine treatment resulted in the accumulation of autophagosomes (white arrow Fig. 2B), but it did not entirely block the fusion between autophagosome and the lysosome as indicated by LC3B and LAMP2 partial co-localization (Fig. 2B). On the other hand, LC3A and LC3B did not co-localize thus supporting different autophagosome formation in SH-SY5Y cells (Figure S2C). Moreover, LAMP2 and LC3B did not co-localize in serum-starved and chloroquine treated SH-SY5Y cells, hence supporting a complete block in autophagy flux (Figure S2C). To further confirm the immunofluorescence analysis, flow cytometry was used to monitor autophagy flux in both lines. CCHE-45 treatment with rapamycin resulted in higher mean fluorescent intensity (MFI) compared to stained control (p-value = 0.047). Compared to SH-SY5Y, CCHE-45 displayed significantly higher levels of MFI in DMSO (p-value = 0.001), and rapamycin (p-value = 0.049). Similarly, MFI was higher in CCHE-45 upon treatment with both rapamycin and chloroquine (p-value = 0.042) (Fig. 2C). Chloroquine treatment did not significantly affect CCHE-45 cells compared to rapamycin treatment alone (p-value = 0.8) (Figure S2C). In contrast, SH-SY5Y cells showed a significantly increased MFI when treated with rapamycin, and rapamycin plus chloroquine (p-value = 0.005 and 0.012 respectively) (Fig. 2C). Examining the soluble and the insoluble protein fractions from CCHE-45 cells, vimentin was found in the insoluble fraction under control conditions as well as after 2 hours of serum starvation, while after 6 hours the majority of vimentin was found in the soluble fraction (Fig. 2D). These results suggest that aggresomes may be disassembled rather than degraded upon induction of autophagy. β-actin on the other hand, was primarily found in the soluble fraction (Fig. 2D).

### Intergenic CpG island methylation silences *LC3A-V1* expression in choroid plexus carcinoma tumors

**Figure 3:**
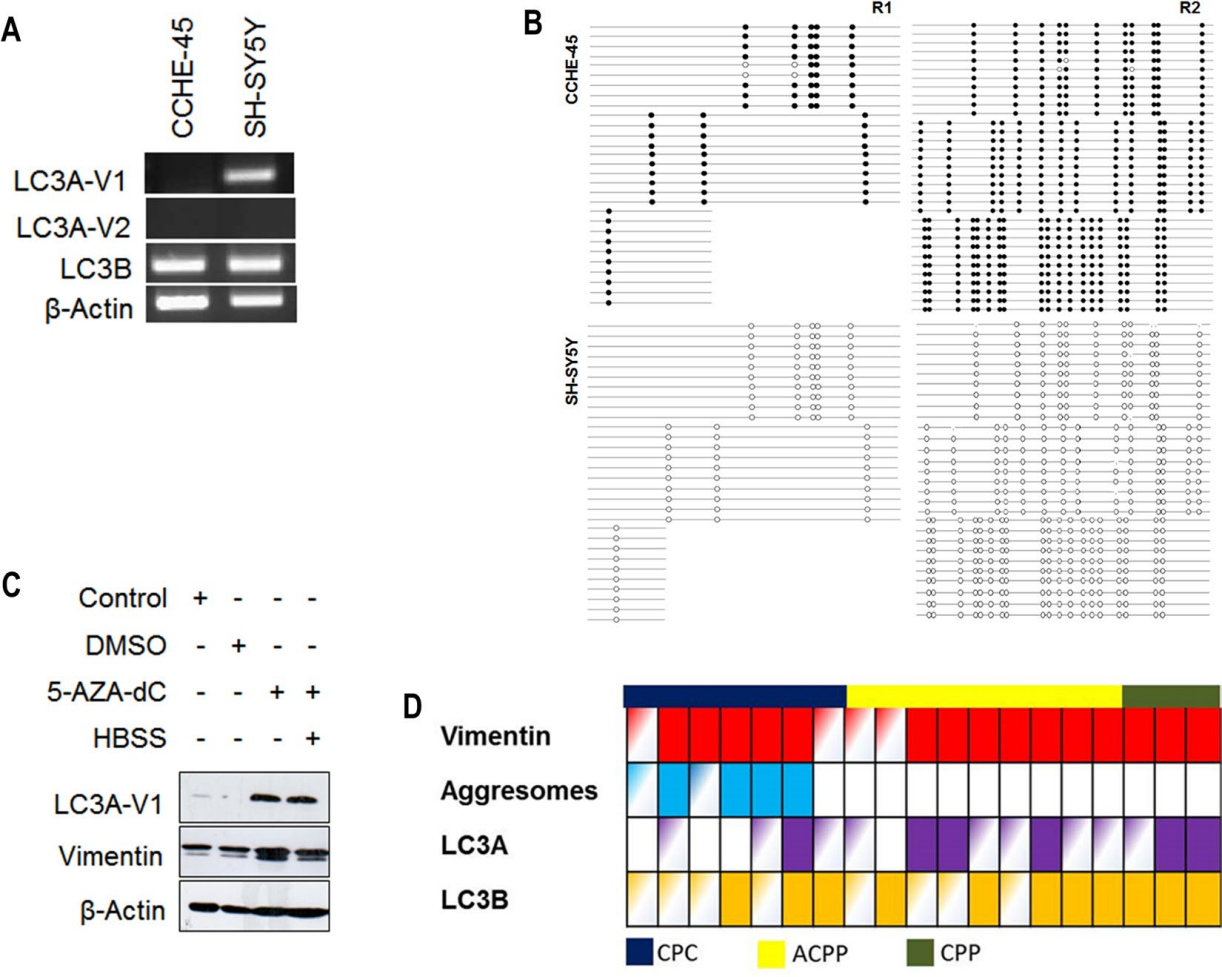
Inactivation of *LC3A* expression in choroid plexus carcinoma. (**A**) Expression of *LC3A-V1*, *LC3A-V2* and *LC3B* were determined using RT-PCR in CCHE-45 and SH-SY5Y cell lines. β-actin was used as an internal control. (**B**) Bisulfite modified DNA PCR products from CCHE-45 and SH-SY5Y. Primers were designed to amplify CpG island (R1 and R2) upstream of *LC3A-V1* using bisulfite sequencing. Diagrams show methylated CG dinucleotide in CCHE-45 R1 and R2 with no methylation detected in SH-SY5Y. (**C**) Western blot analysis of CCHE-45 cells treated with 10 µM 5-AZA-dC for 4 days then serum starved in HBSS for 2 hours. LC3A protein was restored following 5-AZA-dC treatment. No change in vimentin protein levels was detected. (**D**) Schematic representation for immunostaining of 19 cases CPC (blue), ACPP (yellow) or CPP (olive). FFPE tissue sections were stained with vimentin, LC3A or LC3B. Solid, partial and clear color indicates positive, focal or negative stain respectively.

Deregulation of signaling pathways and microtubule-associated proteins were shown to correlate with clinical outcomes in some tumors^19–21^. To verify that LC3A is expressed in normal brain tissue, the expression of LC3A and LC3B was examined using BrainSpan transcriptome datasets. LC3A and LC3B were expressed in all brain regions and during different developmental stages (Figure S3A). *LC3A* has two transcriptional variants that differ in transcription start site (Figure S3B). Both *LC3A* variants were not expressed in CCHE-45 cells, while only *LC3A-V1* was expressed in SH-SY5Y cells (Fig. 3A). Moreover, the expression of *LC3B* and the absence of *LC3A* in the parent tumor tissue supports the inactivation of the *LC3A* (Figure S3C). *LC3A-V1* gene expression was previously reported to be silenced by protmoter methylation in a wide range of tumors^20, 22^. Concordant with these data, loss of *LC3A-V1* expression in CCHE-45 cells was due to intergenic CpG island methylation (Fig. 3B) which was restored upon 5AZA-dC treatment (Fig. 3C). Bisulfite sequencing of CCHE-45 parent tumor indicated complete promoter methylation in all ten clones examined (Figure S3D). The expression of LC3A and LC3B ﻿was assessed in 19 formalin-fixed paraffin-embedded (FFPE) CPT samples using immunohistochemical analysis. While LC3B was detected in all tumors, LC3A positive stain was present in CPP and either focal or absent in both ACPP and CPC (Fig. 3D).

### Re-expression of LC3A resolves aggresome vimentin cage

**Figure 4:**
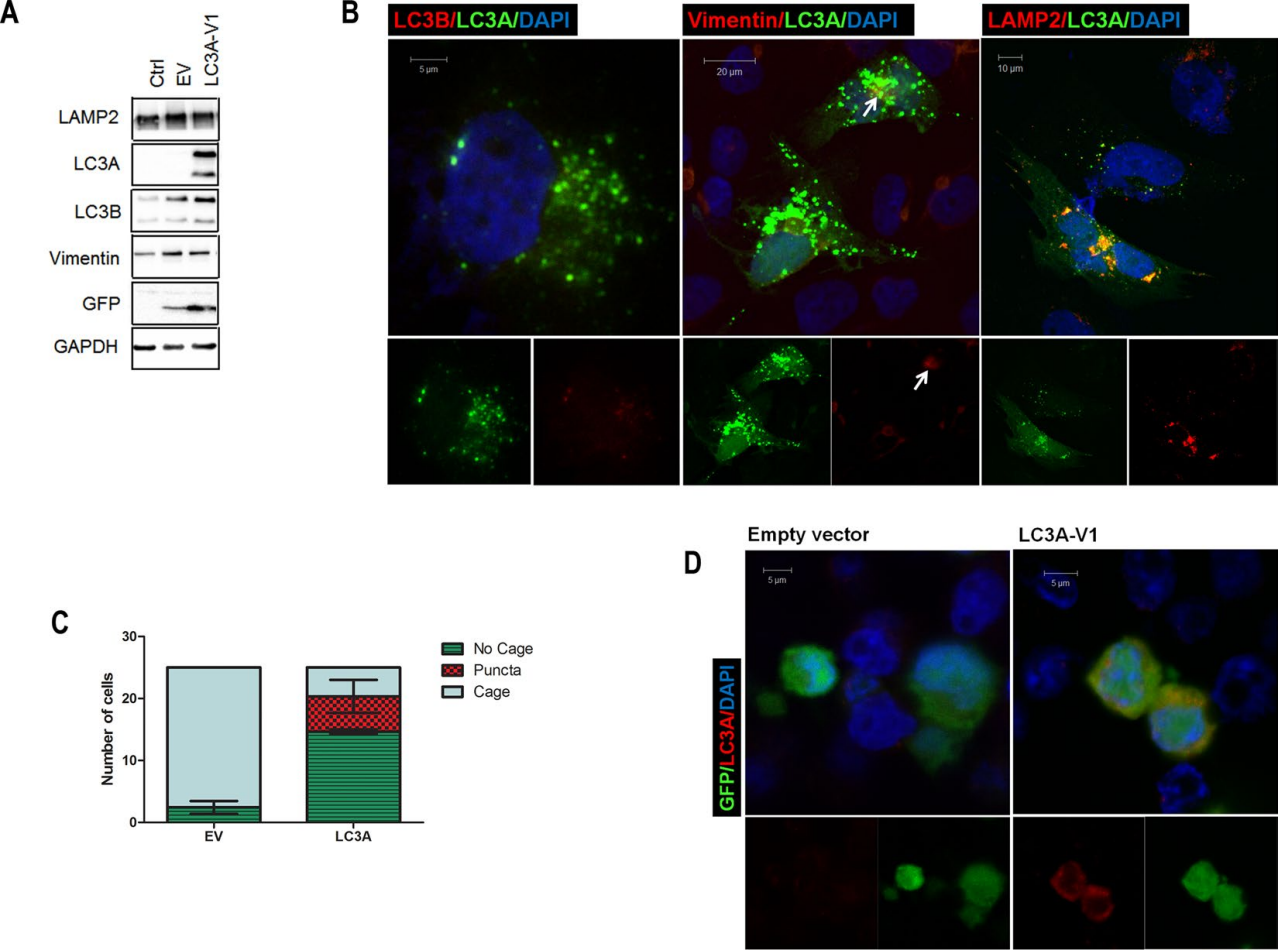
Expression of LC3A induces the formation of LC3A positive autophagosome. (**A**) Western blot analysis of CCHE-45 transfected with empty vector or LC3A-V1 for 48 hours. GAPDH was used as a loading control. (**B**) Immunofluorescence analysis of CCHE-45 cells transfected with empty vector or LC3A-V1. Cells were co-immunostained with mouse anti-vimentin (red) and rabbit anti-LC3A (green), mouse anti-LAMP2 (red) and rabbit anti-LC3A (green) or mouse anti-LC3B (red) and rabbit anti-LC3A (green). Staining was visualized using Alexa Fluor 488 goat anti-rabbit antibody and Alexa Fluor 555 goat anti-mouse. (**C**) Quantification of aggresomes vimentin cage following LC3A-V1 transfection. Vimentin cage structures were counted manually in GFP positive cells. LC3A-V1 transfected cells GFP positive were also examined for LC3A puncta. Bars represent the average value from three independent experiments. Error bars represent ± SEM. (**D**) Immunofluorescence analysis of HEK293-T cells transfected with empty vector or LC3A. LC3A expression was examined using rabbit anti-LC3A (red) in GFP positive cells. EV = empty vector, Ctrl = control non- transfected cells.

The correlation between the presence of aggresomes and *LC3A*-V1 silencing in CCHE-45 and CPC tumors, suggested that the lack of *LC3A*-V1 expression in CPC may affect the accumulation of aggresomes. To elucidate the role of LC3A, we cloned *LC3A-V1* cDNA downstream of a CMV promoter and transiently transfected CCHE-45 cells with p-IRES2-AcGFP (hereafter empty vector) or p-IRES2-AcGFP-LC3A-V1 (hereafter LC3A-V1). The expression of GFP and LC3A in CCHE-45 cells was confirmed by immunoblot analysis (Fig. 4A). Interestingly, LC3A-V1 transfected cells displayed cytoplasmic aggregates which surrounded the aggresomes and co-localized with LAMP2, with no visible localization with LC3B (Fig. 4B). Importantly, aggresomes’ vimentin cage was either resolved, or formed a localized cluster rather than a cage in LC3A transfected cells (Fig. 4B). A total of 25 GFP positive cells were counted in empty vector and LC3A-V1 transfected cells, and vimentin cage was compared in both cell populations. The majority of LC3A-V1 transfected cells, with LC3A positive puncta, displayed resolved vimentin cage compared to empty vector transfected cells (p value = 0.0004) (Fig. 4C). To ensure that the observed LC3A aggregates are not due to GFP aggregation, CCHE-45 cells transfected with empty vector or LC3A-V1 were fixed in 4% paraformaldehyde and GFP was examined alone or with each antibody individually (Figure S4A). LC3A positive aggregates were detected in LC3A transfected cell only when anti-LC3A antibody was used (Figure S4A, white arrows). To further determine if LC3A puncta formation was a consequence of aggresomes presence, HEK293-T cells which do not express LC3A were transfected with empty vector or LC3A-V1 for 48 hours. Immunoblot analysis confirmed the expression of both GFP and LC3A (Figure S4B). No cytoplasmic puncta were observed in HEK293-T LC3A-V1 transfected cells (Fig. 4D). Together, these results suggest LC3A re-expression in CCHE-45 leads to autophagosomes formation, which could potentially alter aggresomes’ vimentin cage. While we did not identify the reason for aggresomes formation in CCHE-45 cells, the co-localization of LC3A with LAMP2 supports autophagy activation under basal conditions.

## Discussion

The exact molecular mechanisms for CPC pathogenesis remain largely uncharacterized. The best defined mechanism involved in CPC is related to the dysfunction of the tumor suppressor gene *TP 53* ^23^. Recent reports have further implicated aberrant notch signaling^24^, *TAF12*, *NFYC* and *RAD54L* as oncogenes driving CPC tumors^25^. The rarity of CPC and the lack of tumor models for the disease are two major obstacles to sufficiently understand their etiology. The development of CCHE-45 provides a new cell line to the very limited CPC repository. We describe aggresome formation in CPC as one mechanism through which these tumors could potentially overcome proteotoxic stress. Aggresomes’ presence in CPC but not in ACPP and in CPP supports the notion that aggresome accumulation is more likely to be associated with more aggressive tumors. While we did not identify the trigger for aggresome formation in CCHE-45 cells, we observed changes in the number of chromosomes, which could potentially contribute to the altered protein levels. To sustain cellular homeostasis, it is essential for CCHE-45 cells to maintain aggresomes. This was supported by the significant decrease in cell proliferation upon HDAC6 inhibition.

The role of autophagy in cancer has been controversial. In RAS driven tumors, cancer cells were found to rely on autophagy to accommodate high metabolic demand hence promoting cell survival^26, 27^. In contrast, mutation in *BECN1* gene was found to be monoallelecally inactivated in breast, ovarian and prostate cancers^28^. Furthermore, *Beclin1* ^+/−^ mice are prone to tumor development, supporting tumor suppressor role for autophagy^29, 30^. Because of the debated role of autophagy, it is often described as a double edged sword, where promotion of cell survival or activation of apoptosis is based on the cellular context. Part of the controversy on the role of autophagy is attributed to how it is monitored. The conventional marker for autophagy is LC3B protein. However, recent reports indicate that LC3B contributes to one type of autophagosome induced under stress, which is different from LC3A positive autophagosome^31^. Our data demonstrates that autophagy flux is enhanced at basal conditions and under stress in CCHE-45 cells. The inability to block autophagy flux by chloroquine supports the hypothesis that upon autophagy activation, aggresomes’ inducers may be reduced. Consequently, autophagy flux is maintained in order to achieve cellular homeostasis. Induction of nonselective macroautophagy, results in altered assembly of the vimentin cage rather than removal of aggresomes by engulfment. This notion is further supported by TEM for CCHE-45 cells, which showed autophagic vacuoles present in close proximity to aggresomes. These results are in line with previous reports for autophagic vacuoles and lysosome recruitment to aggresomes to facilitate their degradation following proteasome inhibition^32, 33^.

Expression pattern of LC3A was investigated in several studies in correlation with patient clinical outcome^34–36^. Three different patterns were identified, diffuse cytoplasmic, juxta nuclear or stone like structure, where the latter is correlated with poor prognosis^34–36^. Moreover, LC3A was found to be transcriptionally silenced by methylation in esophageal squamous cell carcinoma cell lines and its re-expression resulted in decreased *in vivo* tumor growth, hence suggesting a tumor suppressor role^20^. Our study further supports a cellular protective role for LC3A protein. Under basal conditions, aggresomes’ vimentin cage may be maintained through LC3A gene silencing. The disassembly of the vimentin cage following LC3A-V1 expression, coupled with recruitment of LAMP2 independent of LC3B, suggests that autophagy flux is activated independent of macroautophagy. In line with previous reports, two different autophagosomes; LC3B mediated under stress and LC3A mediated under basal condition.

Inactivation of *LC3A* expression was reported in several tumors^20^; however it was coupled with aggresomes formation only in multiple myeloma^18^. Our data suggests that *LC3A* gene silencing is an initial event prior to aggresome formation. The re-expression of *LC3A* is proposed to alter the autophagy basal flux which may lead to the clearance of aggresomes inducers resulting in vimentin cage resolution. It may also be disrupting a specific protein complex that may be integral for vimentin cage assembly. Since the alteration of vimentin cage by HDAC6 inhibition results in significant cell death, by the same notion LC3A expression could potentially lead to reduced cell survival. Therefore, aggresomes may be a potential new target for the treatment of CPC and other tumors with similar phenotype.

## Methods

### Specimen Collection and Establishment of CCHE-45 Cell Line

Tumor diagnosis was carried out at the Department of Pathology, Children’s Cancer Hospital Egypt 57357 (CCHE). Patients under 18 years of age diagnosed CPC, CPP or ACPP with no prior exposure to radiotherapy or chemotherapy treatment were enrolled in the study. The generation of the choroid plexus carcinoma cell line was performed as described previously^37^. The study protocol for the generation of the cell line was approved by the Children’s Cancer Hospital Institutional Review Board (IRB). Accordingly, informed consent was obtained from participants’ legal guardians. CCHE-45 cell line was authenticated using Multiplex Cell Authentication by Multiplexion (Heidelberg, Germany)^38^. The single nucleotide polymorphism (SNP) profiles for the cells and the parent tumor matched and were unique. CCHE-45 cells were maintained in RPMI (Lonza) supplemented with 10% FBS (Lonza).

### Karyotype and FISH Analysis

Metaphase preparations were obtained from cell lines according to standard cytogenetics procedures. Giemsa staining and clonal chromosomal abnormalities were described according to the International System for Human Cytogenetic Nomenclature. FISH was performed on metaphase preparations from the same culture passage as conventional karyotyping. Whole chromosome paint and TP53/D17Z1 probes were used according to the manufacturers’ instructions (Metasystems, and Abbott). The slides were analysed using Leica DM5500 B microscope (Leica Microsystems); subsequently image acquisition using JAI video camera and image analyzer system (Applied Imaging Ltd) were used.

### Cell lines, Induction of Autophagy and Drug Treatment

Neuroblastoma SH-SY5Y cell line is a kind gift from Dr. Juma Mora at Sant Joan De Deu, Barcelona, Spain. SH-SY5Y cells were authenticated using AmpFlSTR® SGM Plus® PCR Amplification Kit (Applied BioSystems). SH-SY5Y and HEK293-T cells were cultured in RPMI and DMEM (Lonza) respectively supplemented with 10% FBS (Lonza). For induction of autophagy, cells were serum starved in Hank’s balanced salt solution (HBSS) (Lonza) for 2 and 6 hours. For HDAC6 inhibition, cells were treated with 20 μM tubacin or niltubacin (Enzo Life Sciences). For *5-aza*-2′-deoxycytidine (5-AZA-dC) treatment (Sigma Aldrich), cells were treated with either DMSO or 10 μM 5-AZA-dC for four successive days.

### Western Blot Analysis

The preparation of whole-cell lysates and the isolation of soluble and insoluble protein fractions were performed as previously described^39^. The following primary antibodies were used with (1:1000) dilutions: rabbit anti-LC3A (Cat # 4599, Cell Signaling Technology), rabbit anti-LC3B (Cat # 3868, Cell Signaling Technology), mouse anti-acetylated α-tubulin (Cat # sc-23950, Santa Cruz Biotechnology), mouse anti-LAMP2 (Cat # sc-18822, Santa Cruz Biotechnology), rabbit anti-vimentin (Cat # 5741, Cell Signaling Technology), rabbit anti- GAPDH (Cat #5174S, Cell Signaling Technology) and mouse anti-β-Actin (Cat # 3700, Cell Signaling Technology) followed by secondary anti-mouse (1:5000) or anti-rabbit (1:5000) antibody then washed and visualized using ECL Chemiluminescence Western blot substrate (ThermoScientific).

### Immunostaining and Immunofluorescence

Automated immunostaining was carried out using Ventana BenchMarkXT platform (Ventana). The following antibodies were used; anti-vimentin and anti-cytokeratin (Ventana), rabbit anti-LC3A and rabbit anti-LC3B. Immunofluorescence was performed as described previously^40^. For immunofluorescence, the following primary antibodies were used at the indicated concentrations: rabbit anti-LC3A (1:50) (Cat # 4599, Cell Signaling Technology), rabbit anti-LC3B (1:50) (Cat # 3868, Cell Signaling Technology), rabbit anti-vimentin (1:50) (Cat # 5741, Cell Signaling Technology), mouse anti-vimentin (1:100) (Cat # ab8978, Abcam), mouse anti-LAMP2 (1:50) (Cat # sc-18822, Santa Cruz Biotechnology), and mouse anti-LC3B (1:50) (Cat # sc-271625, Santa Cruz Biotechnology). Bound antibodies were visualized using Alexa Fluor 555 or Alexa Fluor 488 secondary antibodies (1:500) (Cell Signaling Technology). Cells were then counterstained using 4, 6-diamidinophenylindole (DAPI). Images were acquired using LSM 710 confocal scanning laser microscope (Carl Zeiss).

### Transmission Electron Microscopy (TEM)

Cell processing and TEM imaging was performed at Cairo University Research Park. In brief, cells were fixed with glutaraldehyde and osmium tetroxide, then dehydrated in alcohol and embedded in an epoxy resin. Microtome sections were prepared at approximately 500–1000 µm thickness with a Leica Ultracut UCT ultramicrotome (Leica Microsystems). Thin sections were stained with tolodin blue (1X). Ultrathin sections were prepared at approximately 75–90 µm thickness and stained with uranyl acetate and lead citrate. Sections were examined using JEM-1400 TEM (JEOL) and captured by CCD camera AMT, optronics camera with 1632 × 1632 pixel format as side mount configuration.

### Cell Viability Assay

Fifty microliters of cell culture medium were added per well to 96-well electronic microtiter plate (E-Plate) for impedance background measurement. CCHE-45 cells were then plated at 12000 cells/well in 24 hours prior to treatment. The E-Plate was incubated at 37 °C with 5% CO_2_ and monitored on the real time cell analysis xCELLigence (ACEA Biosciences) at 5-minute time intervals. The next day, cells were treated with different concentrations of tubacin or niltubacin (Enzo Life Science). Cells were monitored for up to 72 hours post treatment. Cell index (CI) was plotted against different concentrations of tubacin or niltubacin. Experiment was performed three times and three wells/drug concentration were used.

### Constructs, RT-PCR, Cloning and Expression of LC3A Gene in CCHE-45

RNA isolation was performed using TRizol Reagent (Invitrogen) according to manufacturer’s instruction. LC3A-V1 was amplified by polymerase chain reaction (PCR) from SH-SY5Y cells using primers described in Table 1 and cloned in p-IRES2-AcGFP plasmid (Clonetech) using *EcoRI* and *BamHI* restriction enzymes. Positive colonies were sequenced to confirm correct sequences for LC3A-V1 (NM_032514.3).

**Table 1:** Primer sequences for expression, cloning and bisulfite sequencing analysis.

Primer Name	Sequence	Product Size in bp
**LC3A_V1_F**	5′-CCTCAGACCGGCCTTTCAA-3′	1126
**LC3A_V1_R**	5′-AGCTGCTTCTCACCCTTGTA -3′	
**LC3A_V2_F**	5′-ACTCCTGACTGCATGGAAGC-3′	172
**LC3A_V2_R**	5′-GTCCACAGCTGCTTTTCCAC-3′	
**LC3B_F**	5′-CGGAGAAGACCTTCAAGCAG-3′	168
**LC3B_R**	5′-TGACATGGTCAGGTACAAGGA-3′	
**LC3A_region1_F**	5′-ATTTTTGGTAGTTTTTTTTTAGG-3′	313
**LC3A_region1_R**	5′-ACAATCAAACACAAAATAAAACA-3′	
**LC3A_region 2_F**	5′-GTTTTATTTTGTGTTTGATTGTG-3′	421
**LC3A_region 2_R**	5′-TCACAACATTCCTTAAAAAAAA-3′	
**β-Actin_F**	5′-CTGAAGTACCCCATCGAGCA-3′	215
**β-Actin_R**	5′-AGCCTGGATAGCAACGTACA-3′	
**LC3A_V1_cDNA_F**	5′-AAAAAGAATTCATGCCCTCAGACCGGCC-3′	366
**LC3A_V1_cDNA_R**	5′-AAAAAGGATCCTCAGAAGCCGAAGGTTTCC-3′	

### Bisulfite Sequencing

DNA bisulfite modification and sequencing were performed as described previously^41^. In brief, DNA was extracted from CCHE-45, SH-SY5Y and CPC FFPE tissue sample. One µg of DNA was used for bisulfite DNA modification using EpiTect Bisulfite DNA conversion kit (Qiagen) according to the manufacturer’s protocol. Primer sequences for bisulfite sequencing were designed using MethPrimer Software (Applied BioSystems) described in Table 1. For bisulfite sequencing, PCR products were then extracted from agarose gel, tailed, purified, cloned into pTZ57R/T vector using InsTAclone PCR Cloning Kit (ThermoScientific) and transformed into *E-coli* JM109 competent cells (TaKaRa). Cloned fragments from positive colonies were used as template for sequencing with the BigDye Terminator v3.1 Cycle Sequencing protocol (Applied BioSystems) on the ABI 3130 DNA Analyzer to identify methylated and unmethylated sequences. Sequences from ten clones from each sample were then analyzed using BiQ Analyzer^42^.

### Autophagy Detection by Flow Cytometry

Autophagy detection was performed using CYTO*-*ID^*®*^ Autophagy Detection Kit *(*Enzo Life Sciences) following the manufacturer’s protocol. In brief, cells were: control untreated or treated with DMSO, 500 nM rapamycin, or 500 nM rapamycin plus 50 µM chloroquine for 18 hours. The next day cells were stained with green detection reagent. Unstained cells were used as negative control. Samples were analyzed in green FL1 channel of flow cytometer (Beckman Coulter Inc). Arithmetic means were then used for analysis. Assay was repeated at least three times.

### BrainSpan Data Analysis

Human BrainSpan project was used to obtain RPKM for the LC3A (ENSG00000101460) and LC3B (ENSG00000140941). Expression values were calculated for each gene according to developmental stage or across different brain regions. Analysis was performed using R statistical language version 3.2.2.

### Statistical Analysis

All experiments were repeated three times. Data are expressed as ± SEM. The significance of difference among means was evaluated using paired t-test. Significant differences were defined as p ≤ 0.05.

All methods indicated in the section above were performed in accordance with the relevant guidelines and regulations.

### Data Availability

All data generated or analysed during this study are included in this published article (and its Supplementary Information files)^43^.

## Electronic supplementary material

Supplementary Data
