# Supplementary material for: LC3A Silencing Hinders Aggresome Vimentin Cage Clearance in Primary Choroid Plexus Carcinoma
Source: Sci Rep. 2017 Aug 14;7:8022. doi: 10.1038/s41598-017-07403-5 (PMC5556083; doi:10.1038/s41598-017-07403-5)
Supplement: Supplementary file 1 — Supplementary Data [file 41598_2017_7403_MOESM1_ESM.pdf]

LC3A Silencing Hinders Aggresome Vimentin Cage Clearance in Primary Choroid Plexus Carcinoma

Authors:

Marwa Nassar [1, 4, 7], Heba Samaha [1, 7], Myret Ghabrial [1], Maha Yehia [2], Hala Taha [2, 6,], Sherin Salem [3, 6], Khaled Shaaban [3, 6], Mariam Omar [1], Nabil Ahmed [1, 5] and Shahenda El-Naggar [1] *

Supplemental Figures:

Figure S1:

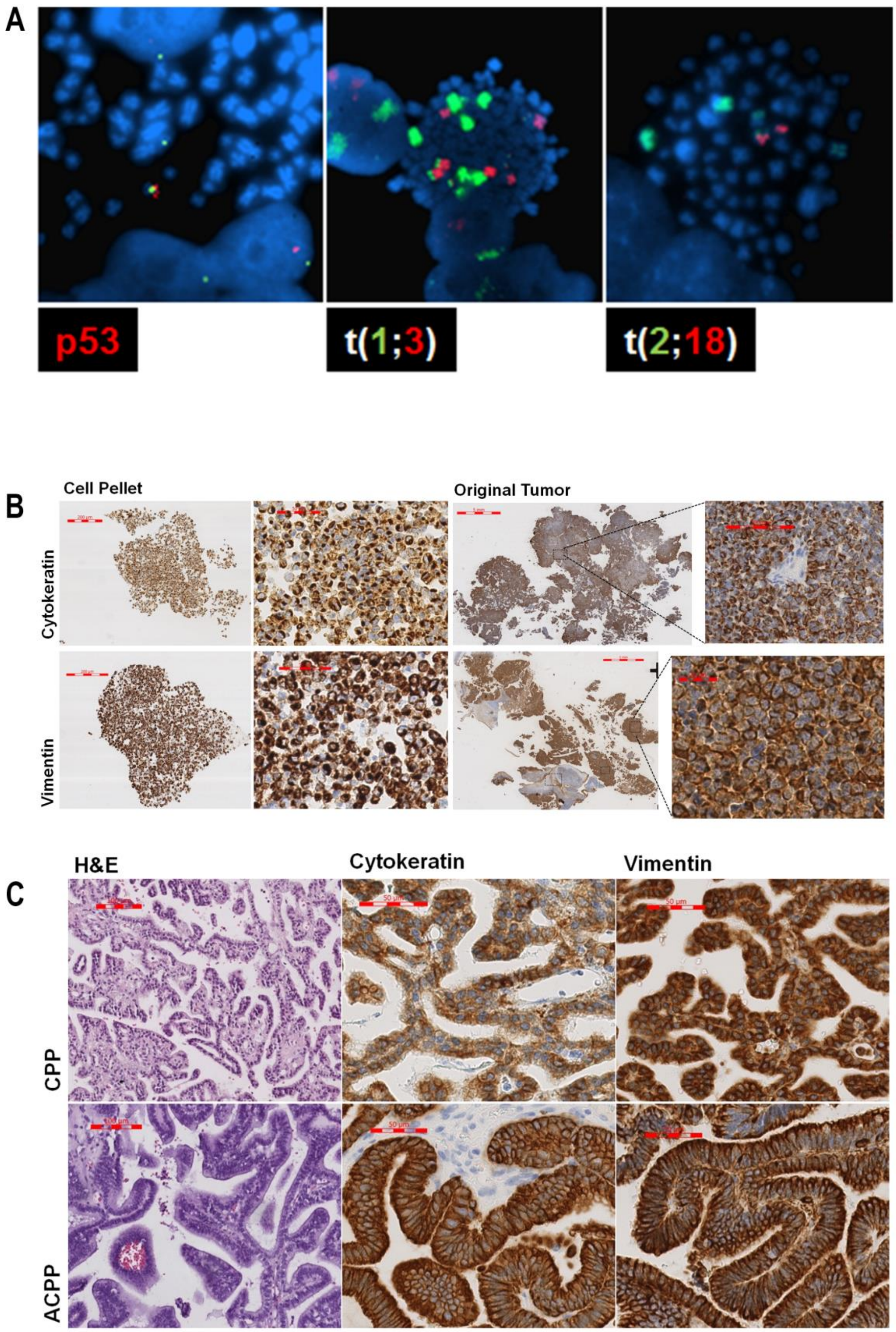

Figure S2:

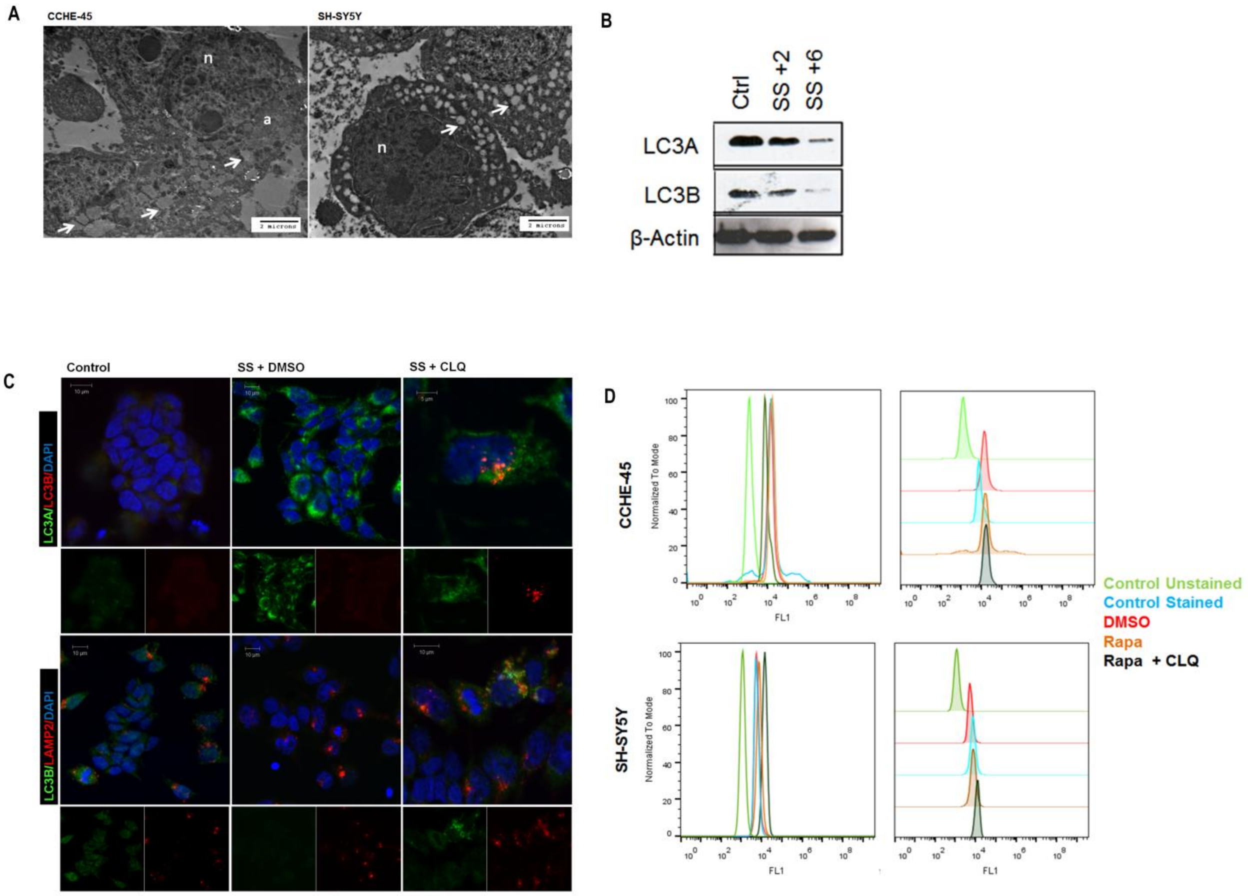
A
CCHE-45
SH-SY5Y
n
a
n
2 microns
2 microns
B
Ctrl
SS +2
SS +6
LC3A
LC3B
β-Actin
C
Control
SS + DMSO
SS + CLQ
LC3A/LC3B/DAPI
LC3B/LAMP2/DAPI
D
CCHE-45
SH-SY5Y
Normalized To Mode
FL1
Control Unstained
Control Stained
DMSO
Rapa
Rapa + CLQ

Figure S3:

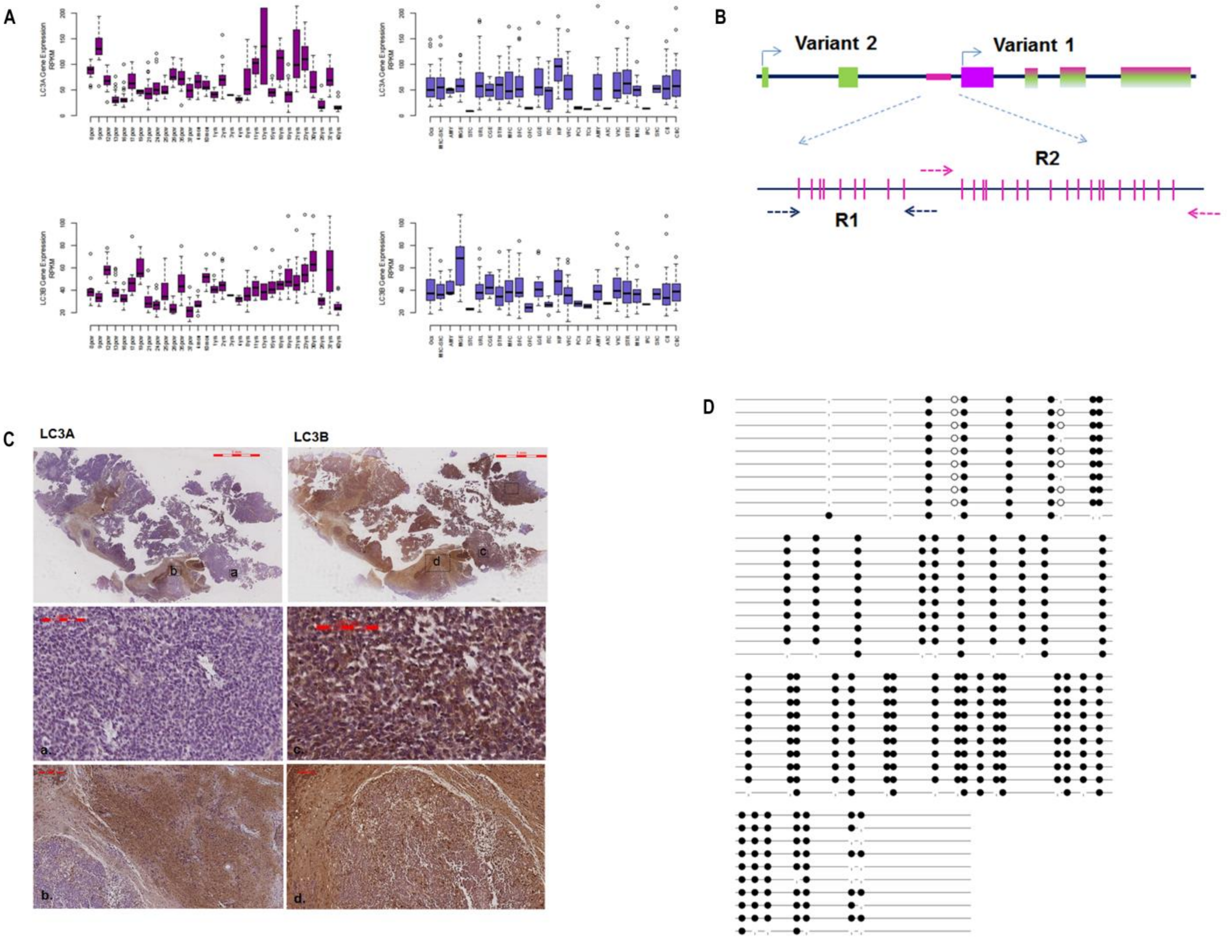

Figure S4:

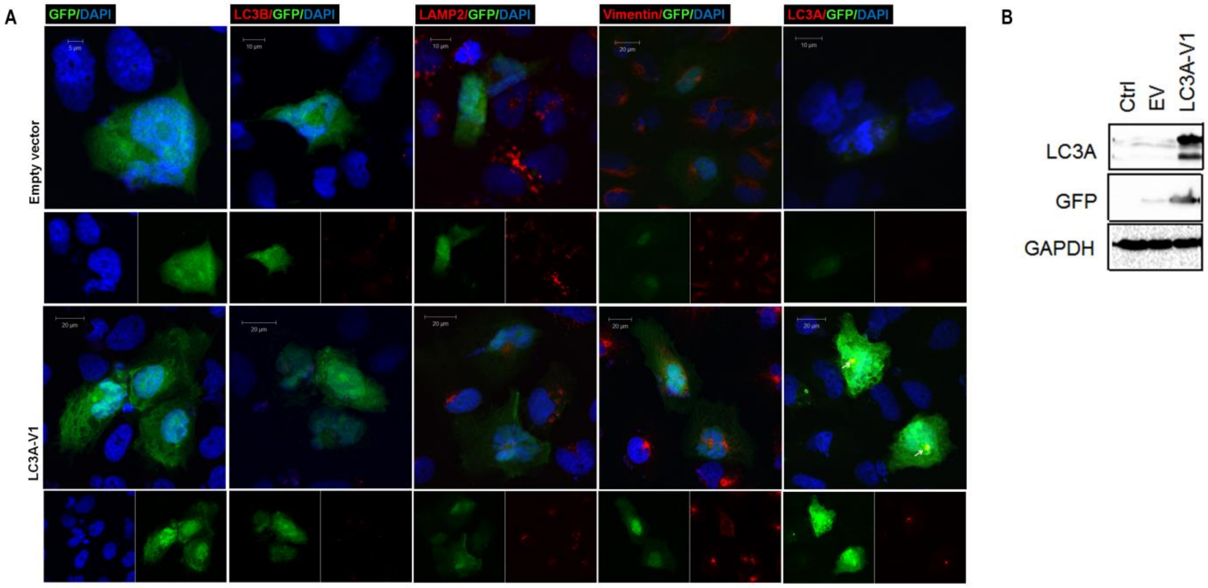
A
GFP/DAPI
LC3B/GFP/DAPI
LAMP2/GFP/DAPI
Vimentin/GFP/DAPI
LC3A/GFP/DAPI
Empty vector
LC3A-V1
B
Ctrl
EV
LC3A-V1
LC3A
GFP
GAPDH

Figure S5:

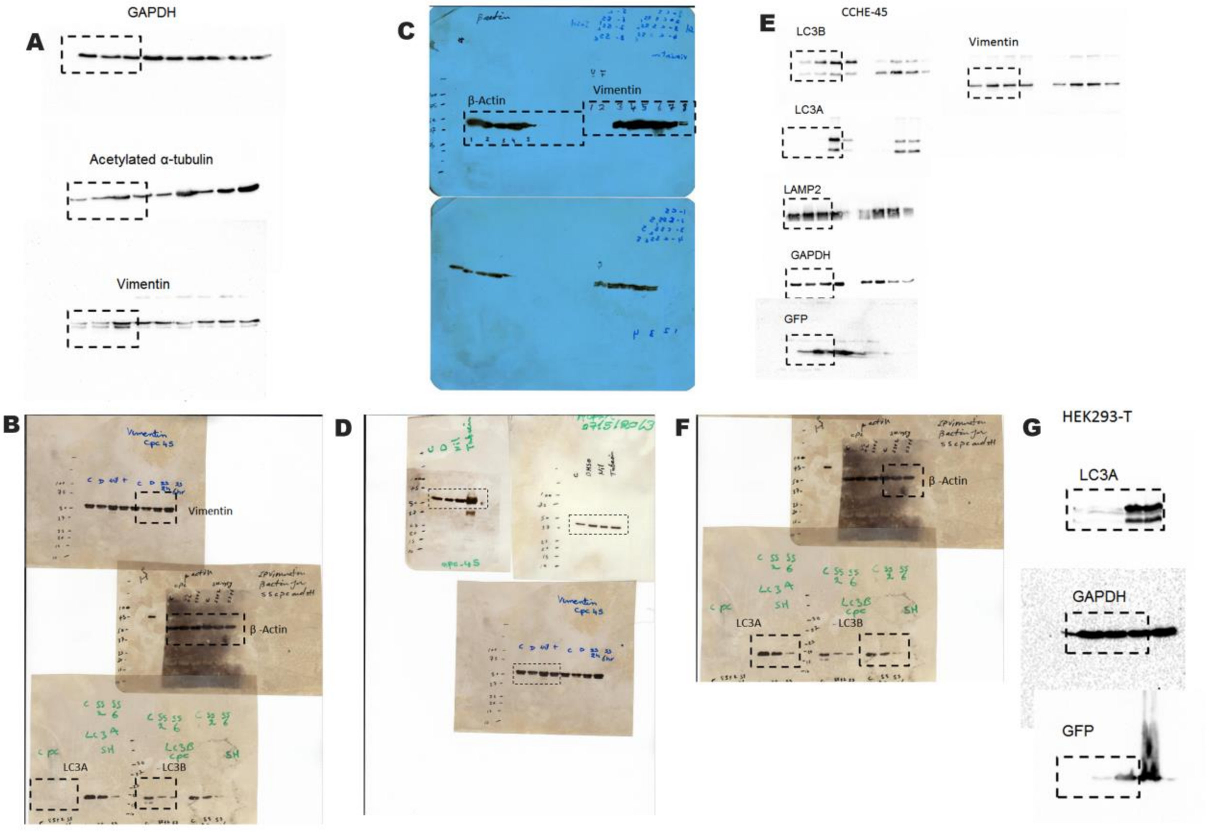
A
GAPDH
Acetylated α-tubulin
Vimentin
C
β-Actin
Vimentin
E
CCHE-45
LC3B
Vimentin
LC3A
LAMP2
GAPDH
GFP
B
Vimentin
β -Actin
LC3A
LC3B
D
F
β -Actin
LC3A
LC3B
G
HEK293-T
LC3A
GAPDH
GFP

Supplemental Figures Legend

Figure S1: Aggresomes detected in CCHE-45 cells are present in the original tumor and absent in both CPP and ACPP.

A. FISH analysis using red and green probes to detect *TP53* gene and centromere respectively, translocation t(1;3) and translocation t(2;18).

B. CCHE-45 cell pellet and the original tumor were immuno-stained with cytokeratin and vimentin indicated by para nuclear localization of vimentin and cytokeratin.

C. CPP and ACPP FFPE tissue hematoxylin and eosin (H&E) preparation and immunohistochemical analysis for vimentin and cytokeratin (Ventana,Tucson, Arizona, USA). Diffused staining for vimentin and cytokeratin detected in both tumors.

Figure S2: Induction of autophagy in CCHE-45 and SH-SY5Y cells.

A. TEM examination of CCHE-45 and SH-SY5Y cells using JEM-1400 T (JEOL). Cells were serum starved in HBSS for 2 hours before fixation for TEM analysis**.** a = aggresomes, n = nucleus, white arrow = autophagic vacuoles.

B. Western blot analysis of SH-SY5Y cells. Cells were left untreated (Ctrl) or serum starved in HBSS for 2 and 6 hours.

C. Immunostaining of SH-SY5Y cells cultured under normal conditions or serum starved in HBSS for 5 hours or serum starved and treated with 50 µM chloroquine. Cells were fixed, and immunostained with mouse anti-LC3B and rabbit anti-LC3A, mouse anti-LAMP2 and rabbit anti-LC3A or rabbit anti-LC3B and mouse anti-LAMP2 antibodies. Cells were visualized using Alexa Fluor 488 goat anti-rabbit antibody or Alexa Fluor 555 goat anti-mouse. DAPI was used as counterstain to visualize the nucleus. SS = serum starved, CLQ = chloroquine

D. Flow cytometry-based profiling of CYTO-ID Autophagy detection in CCHE-45 and SH-SY5Y cells. Cells were untreated or treated with 500 nM rapamycin, 50 μM chloroquine or both for 18 hours. Results are presented as histogram overlay. All experiments were performed three times. Rapa = rapamycin, CLQ = chloroquine.

Figure S3: Silencing of LC3A expression in CPC tumors

A. BrainSpan data analysis. *LC3A* and *LC3B* gene expression as a function of different developmental stages (upper panel) and as a function of different regions of the brain (lower panel) using RNA-Seq data from the BrainSpan Project. Expression of *LC3A* and *LC3B* was detected in all developmental stages and in different parts of the brain. pcw = post conception weeks, mon = month, yrs = years, Ocx = occipital neocortex, M1C-S1C = primary motor-sensory cortex, AMY = amygdaloid complex, MGE = medial ganglionic somatosensory cortex, CB = cerebellum, CBC = cerebellar cortex , MD = mediodorsal nucleus of thalamus.

B. Schematic representation of *LC3A* exon-intron structure showing the difference in transcriptional start site between *LC3A-V1* and *LC3A-V2*.

C. Immunohistochemical analysis of LC3A and LC3B in CCHE-45 original tumor. LC3A was detected in normal brain tissue (region a) while tumor area (region b) was negative for LC3A expression. On the other hand LC3B expression was detected in normal and tumor tissue region (c and d) respectively.

D. Bisulfite sequencing for CCHE-45 original tumor. Region 2 was amplified using R2 primer set. Methylated CG dinucleotides are indicated by black circles.

Figure S4: LC3A-V1 expression in CCHE-45 and HEK293-T cells.

A. Immunofluorescence analysis of CCHE-45 cells transfected for 48 hours with empty vector or LC3A-V1. Cells were stained with rabbit anti-LC3A, mouse anti-LC3B, rabbit anti-vimentin or mouse anti-LAMP2. Bound antibodies were visualized using Alexa Fluor 555 goat anti-mouse or Alexa Fluor 555 goat anti-rabbit. DAPI was used to visualize the nucleus. .GFP protein was visualized without staining.

B. Western blot analysis for HEK293-T cells transfected with empty vector or LC3A-V1. GFP expression was only detected in transfected cells. GAPDH was used as loading control. EV = empty vector, Ctrl = control.

Figure S5: Western blots scans

Scanned Western blots; A = figure 1C, B = figure 2A, C = figure 2D, D = figure 3C, E = figure 4A, F = figure 2SB, G = Figure 4SB.
